# Validation of insulin-like growth factor-1 as a prognostic parameter in patients with hepatocellular carcinoma in a European cohort

**DOI:** 10.1186/s12885-018-4677-y

**Published:** 2018-07-31

**Authors:** Yvonne Huber, Franziska Bierling, Christian Labenz, Sandra Koch, Irene Schmidtmann, Roman Kloeckner, Sebastian Schotten, Tobias Huber, Hauke Lang, Marcus A. Woerns, Peter R. Galle, Arndt Weinmann, Julia Weinmann-Menke

**Affiliations:** 1 Clinical Registry Unit (CRU), University Medical Center of the Johannes Gutenberg University Mainz, Mainz, Germany; 2 Department of Internal Medicine I, University Medical Center of the Johannes Gutenberg University Mainz, Mainz, Germany; 3 Institute of Medical Biostatistics, Epidemiology and Informatics, University Medical Center of the Johannes Gutenberg University Mainz, Mainz, Germany; 4 Department of Diagnostic and Interventional Radiology, University Medical Center of the Johannes Gutenberg University Mainz, Mainz, Germany; 5 Department of General, Visceral and Transplant Surgery, University Medical Center of the Johannes Gutenberg University Mainz, Mainz, Germany

**Keywords:** Hepatocellular carcinoma, HCC, Overall survival, Clinical database, IGF-1

## Abstract

**Background:**

In hepatocellular carcinoma (HCC), the third leading cause of cancer-related mortality worldwide, the Child-Turcotte-Pugh score (CTP) is one of the most established tools to assess hepatic reserve and determine survival. Serum levels of insulin-like growth factor-1 (IGF-1) are decreased in patients with chronic liver disease or HCC. A modified score combining circulating IGF-1 with the CTP score (IGF-CTP) was recently proposed.

**Methods:**

IGF-CTP scoring was evaluated in 216 patients diagnosed with HCC between 2007 and 2017 to assess the predictive value of serum IGF-1 levels for patient risk stratification and overall survival (OS).

**Results:**

Liver cirrhosis was identified in 80.1% of the study cohort, and alcohol-induced liver disease was the most frequent underlying cause of HCC (44.4%). Serum IGF-1 levels were significantly lower in patients with HCC in cirrhosis compared with non-cirrhotic HCC (*p* < 0.01). A lower serum level of IGF-1 was associated with more advanced stages of liver cirrhosis (*p* < 0.05) and cancer stages (*p* < 0.001). Median OS in the cohort was 11.4 months (range 0.5–118.2 months). OS was significantly higher (10.9 vs. 7.9 months; p < 0.05) in patients with a serum IGF-1 level above the median of 43.4 ng/mL. Patient reassignment using IGF-CTP scoring reclassified 35.6% of patients. Through reassignment, stratification regarding OS was comparable to CTP.

**Conclusions:**

This study is the first to investigate IGF-1 and the IGF-CTP classification in a European cohort of HCC patients. Serum IGF-1 correlates with OS in patients with HCC. However, the IGF-CTP classification was not superior compared to CTP score regarding OS.

## Background

Hepatocellular carcinoma (HCC) is the fifth most common cancer and the third leading cause of cancer-related mortality worldwide [1]. Despite improvements in screening and surgical techniques, as well as the development of non-surgical treatments such as transarterial chemoembolization (TACE) and radiofrequency ablation, the overall prognosis is poor, with a 5-year survival rate of 15% [2]. Treatment decisions for HCC are commonly based on the clinically based Barcelona Clinic Liver Cancer (BCLC) staging system, which classifies patients with HCC into five categories: very early, early, intermediate, advanced, and terminal [3]. BCLC stratifies patients according to performance status, tumor status (tumor size, number of nodules, vascular invasion, extrahepatic spread), and the underlying liver function using Child-Turcotte-Pugh (CTP) score. CTP has become a standard score for assessing hepatic reserve and determining prognosis, as well as survival of HCC [4]. It consists of serum bilirubin, serum albumin, and the international normalized ratio as three objective parameters, along with ascites and encephalopathy as two subjective parameters [5]. However, some limitations of the CTP score have recently been widely discussed. One limitation is the use of subjective variables, which are difficult to assess and susceptible to possibly daily change under the influence of medications and nutritional status. Therefore, other scores were evaluated, like the Model for End-Stage Liver Disease (MELD), which was introduced as a more objective liver score and replaced CTP for stratifying patients for the urgency of liver transplantation [6].

More than 75% of insulin-like growth factor (IGF)-1 is produced by the liver in response to growth hormone from the pituitary [7, 8]. Several studies have demonstrated an association between high circulating IGF-1 levels and increased risk for the development and progression of prostate, breast, and colon cancers [7, 9, 10]. Because the liver produces most of the circulating IGF-1, studies have investigated the link between IGF-1 levels and hepatic function. In patients with chronic liver disease, decreased levels of circulating IGF-1 were found in comparison to healthy controls, leading to the hypothesis that plasma IGF-1 levels reflect hepatic synthetic function and should be considered a surrogate marker for the hepatic reserve [8, 11]. Moreover, Mazziotti et al. demonstrated a link between decreased serum IGF-1 and the development of HCC, which was independent of the grade of hepatic dysfunction [12]. Furthermore, several studies have investigated the use of IGF-1 in HCC patients to correlate HCC progression and survival outcome [13–15]. In these reports, a low baseline serum IGF-1 level was independently associated with reduced overall survival (OS) in patients receiving curative therapy for early stage HCC [16]. To assess hepatic reserve in HCC, Kaseb et al. constructed a modified CTP classification system (IGF-CTP) by replacing the two subjective parameters in the traditional CTP score, ascites and encephalopathy, with the serum IGF-1 level [17] and demonstrated improved OS prediction in HCC patients compared to the CTP score.

The aims of this study were to investigate serum IGF-1 levels as a predictive factor for patient risk stratification and OS as well as the validation of the IGF-CTP classification system in a cohort of European patients with HCC.

## Methods

### Patient characteristics

Patients with confirmed HCC treated at the University Medical Center of the Johannes Gutenberg University Mainz with an initial diagnosis of HCC between January 2007 and January 2017 were included in this retrospective analysis when blood samples and informed consent for IGF-1 analysis was available. The end of follow-up was September 30, 2017. Survival data were acquired from clinical records and by contacting registration offices. The diagnosis of HCC was made according to the AASLD/EASL criteria, and patients were classified using BCLC categories [18]. Tumor differentiation (grading) was classified according to the Edmondson–Steiner classification. Tumor size was documented based on radiological assessment or resected specimen, as applicable, and tumor-specific treatment was extracted from patient records. Liver cirrhosis was determined based on histological confirmation or laboratory results indicating impaired liver function and typical clinical signs including non-malignant ascites, hepatic encephalopathy, thrombocytopenia, splenomegaly, and the presence of esophageal varices. For cirrhotic patients, the CTP score and MELD score were calculated. Etiology of liver disease was evaluated following clinical information, laboratory results, or histological confirmation. Chronic viral hepatitis was diagnosed by a positive test for hepatitis B surface antigen for HBV, and infection with HCV by anti-HCV antibodies (anti-HCV) and HCV-RNA. Alcoholic liver disease was defined by an alcohol consumption of more than 80 g/d in men and 60 g/d in women and the absence of other causes of liver disease. The diagnosis of nonalcoholic steatohepatitis (NASH) was confirmed by typical histological features when biopsy results were available. Cryptogenic cirrhosis in the presence of metabolic risk factors and in the absence of significant alcohol consumption was considered as NASH, as previously established [19]. Diagnosis of primary biliary cirrhosis (PBC) was based on histology or laboratory findings (AMA-M2, elevated immunoglobulin M, pathological alkaline phosphatase (ALP) or gamma-glutamyltransferase (GGT)) while primary sclerosing cholangitis (PSC) diagnosis was based on a typical presentation of bile duct alterations in ERC/MRCP. Hemochromatosis was defined by hemochromatosis gene testing and/or the presence of primary hepatic iron overload. The study was approved by the responsible ethics committee of the Medical Association of Rhineland Palatinate, Mainz, Germany. The study includes data from the doctoral thesis of one of the authors (FB).

### Laboratory parameters and IGF-1 measurement

Laboratory results were obtained at the time of initial HCC diagnosis and considered missing if not available within a period of 90 days. Since stability of IGF-1 has been demonstrated for frozen storage [20], blood samples for IGF-1 measurements were collected and stored at − 80 degrees C until the end of the study. To quantify IGF-1 levels in the circulation, serum samples were analyzed in duplicate using the human IGF-1 Quantikine ELISA (R&D Systems, Cat. No. DG100) kit according to the manufacturer’s instructions.

### IGF-CTP score

The IGF-CTP score replaces the subjective values ascites and encephalopathy from the traditional CTP score with serum IGF-1 levels. It comprises the laboratory values of total bilirubin, albumin, and prothrombin time with identical cut-off points as in the original CTP classification. The new parameter IGF-1 has two cut-off points (26 and 50 ng/mL), which were derived from survival analyses. Serum levels of IGF-1 were scored as 1 point (> 50 ng/mL), 2 points (26 to 50 ng/mL), or 3 points (< 26 ng/mL). Based on the sum of all four laboratory scores, patients can be classified as having class A (4–5 points), B (6–7 points), or C (≥8 points) liver disease [17].

### Statistical analysis

Statistical analyses were done with R version 3.4.2 (www.r-project.org) and GraphPad Prism version 6.0 (GraphPad Software, La Jolla, CA, USA). Data are given as medians and ranges for continuous variables or as absolute and relative frequencies for categorical variables. Comparison of continuous variables was made using the Mann–Whitney U test or Kruskal–Wallis test, respectively. Categorical variables were compared with the Fisher’s exact test or its equivalent for more than two categories. The Kaplan–Meier method was used to create survival curves, whereby survival time was calculated from the time of initial HCC diagnosis. Comparison of survival times was performed with the log-rank test, as was univariate analysis of prognostic variables. A Cox proportional hazards model was used to assess the impact of the reclassification from CTP to IGF-CTP, mimicking the analysis by Kaseb et al. [17]. To compare the prognostic performance of both scores (CTP vs. IGF-CTP), the concordance index (C-index) with concordance index function in package “survcomp” version 1.26.0 was used. The larger the C-index, the more accurate the prognostic prediction. A *p*-value below 0.05 was considered significant.

## Results

### Patient characteristics

From January 2007 to January 2017, a total of 216 patients with an initial diagnosis of HCC were enrolled. We enrolled patients who consented at time of the initial diagnosis to participate in the study and agreed to providing blood samples for further evaluation. The mean age of the study population was 69.6 years (range 25.5–85.0 years), and 86.1% (*n* = 186) were male. Alcohol-induced liver disease was the most frequent underlying cause of HCC in 44.4% (*n* = 96), followed by chronic viral hepatitis (HBV 13.0%, HCV 11.6%) and NASH (8.3%). In 6.5%, the chronic liver disease was cryptogenic, while HCC occurred in 13.4% without underlying liver disease. PBC, PSC, and autoimmune liver disease (AIH) or hemochromatosis were found in 2.8%. Histological data were available in 78.7% of patients. A total of 80.1% of all HCCs developed in a cirrhotic liver. Patient demographics as well as clinical and tumor characteristics at the initial HCC diagnosis are listed in Table 1. The median follow-up time was 8.2 months (range 0.5–120.1 months), without any loss to follow-up.

**Table 1:** Patient demographics and clinical and tumor characteristics at time of initial HCC diagnosis

Characteristics	n (%)
Total patients included	216 (100)
Age at time of diagnosis (y)	69.6 (61.1; 74.1) ^a^
Sex	
Male	186 (86.1)
Female	30 (13.9)
Etiology of liver disease	
Alcohol	96 (44.4)
Viral hepatitis	53 (24.6)
NASH	18 (8.3)
Cryptogenic	14 (6.5)
No liver disease	29 (13.4)
Other	6 (2.8)
Cirrhosis	
Yes	173 (80.1)
No	43 (19.9)
BCLC stage	
BCLC 0	0
BCLC A	36 (16.7)
BCLC B	25 (11.6)
BCLC C	111 (51.4)
BCLC D	44 (20.4)
Tumor grading	
Well	41 (19)
Moderate	75 (34.7)
Poor	38 (17.6)
Missing	62 (28.7)
Tumor size	
≤ 2 cm	15 (6.9)
2–5 cm	78 (36.1)
> 5 cm	82 (38)
Missing	41 (19)
Tumor nodularity	
Solitary	56 (25.9)
Multifocal	108 (50)
Missing	52 (24.1)
Metastasis	
Lymph nodes	19 (8.8)
Distant	24 (11.1)
Missing	173 (80.1)
Intrahepatic vascular invasion	64 (29.6)
Primary therapy	
TACE	87 (40.3)
Resection	27 (12.5)
Liver transplantation	5 (2.3)
Sorafenib	44 (20.4)
Best supportive care	46 (21.3)
Others (SIRT, RFA)	7 (3.2)
Serum α-FP (ng/mL)	46.5 (7.2; 1188.3) ^a^
MELD	10 (7; 13) ^a^
Serum IGF-1	43.4 (27.7; 68.6) ^a^
Overall survival (months)	11.38 (4.6; 33.2) ^a^

^a^Data presented as median and interquartile range (IQR)

Abbreviations: *BCLC* Barcelona Clinic Liver Cancer, *TACE* transarterial chemoembolization, *SIRT* selective internal radiation therapy, *RFA* radiofrequency ablation, *α-FP* α-fetoprotein, *MELD* Model of end stage liver disease, *IGF-1* insulin-like growth factor 1

### Preserved hepatic function in patients with high plasma IGF-1 levels

Median IGF-1 level was 43.4 ng/mL (range 9.6–239.6 ng/mL). There was no difference regarding sex, age, or underlying liver disease. Higher IGF-1 levels were found in HCC developing in non-cirrhotic liver compared to patients with cirrhosis (median (range) 61.1 ng/mL (17.4–230.5 ng/mL) vs. 39.0 ng/mL (9.6–239.6 ng/mL); *p* < 0.01). In patients with normal liver enzymes (alanine transaminase (ALT) ≤40 U/L and aspartate transaminase (AST) ≤45 U/L), IGF-1 levels were significantly increased compared to patients with elevated liver enzymes. In univariate analysis, levels of bilirubin, albumin, international normalized ratio (INR), and platelet counts showed significant correlations with the amount of IGF-1 (Table 2). Correspondingly, the MELD score (all patients: median (range) 10 (6–24)) was associated with lGF-1: MELD score was significantly lower in patients with IGF-1 levels above the median compared to patients with IGF-1 levels below the median (median (range) 8 (6–21) vs. 13 (6–24); *p* < 0.001). Regarding CTP score, IGF-1 levels decreased significantly with more advanced stage of liver cirrhosis (*p* < 0.05). (Table 3). In multivariate analysis, only liver enzymes were significantly associated with the amount of IGF-1 (*p* < 0.01).

**Table 2:** Amount of IGF-1 depending on different characteristics at time of initial HCC diagnosis

Characteristic	Patients, *n* = 216	
	IGF-1 (ng/mL) median (range)	*p*
Age		
≤ 60	37.9 (11.5–230.5)	0.8
> 60	46.6 (9.6–239.6)	
Ethnicity		
White	43.3 (9.6–230.5)	0.3
Other	52.1 (14–239.6)	
Sex		
Male	43.3 (9.6–230.5)	0.4
Female	46.6 (11.8–239.6)	
Cirrhosis		
Yes	39.0 (9.6–239.6)	*< 0.01*
No	61.1 (17.4–230.5)	
BCLC stage		
BCLC A	60.6 (11.6–239.6)	*< 0.001*
BCLC B	57.8 (16–131.8)	
BCLC C	48.2 (10.6–230.5)	
BCLC D	25.8 (9.6–138.8)	
Tumor grading		
Well	40.9 (9.6–239.6)	0.3
Moderate	46.8 (10.6–138.8)	
Poor	55 (10.3–131.8)	
Tumor size		
≤ 5 cm	38.2 (11.6–150.5)	0.2
> 5 cm	47.3 (9.6–239.6)	
Lymph node metastasis		
Yes	58.7 (11.5–230.5)	0.3
No	42 (9.6–239.6)	
Distant metastasis		
Yes	54.2 (13.2–230.5)	0.09
No	42.1 (9.6–239.6)	
Vascular invasion		
Yes	35.8 (9.6–138.8)	*< 0.001*
No	45.8 (11.5–239.6)	
α-FP		
Normal (< 8 ng/mL)	53 (9.6–239.6)	0.08
Elevated (> 8 ng/mL)	41.7 (10.3–138.8)	
ALT		
Normal (≤40 U/L)	52.1 (13.4–239.6)	*0.03*
Elevated (> 40 U/L)	39.2 (9.6–230.5)	
AST		
Normal (≤45 U/L)	68.3 (17.4–150.5)	*< 0.001*
Elevated (> 45 U/L)	39.2 (9.6–239.6)	
Bilirubin		
Normal (≤2 mg/dL)	54.3 (9.6–239.6)	*< 0.00001*
Elevated (> 2 mg/dL)	27.1 (10.3–76.3)	
Albumin		
Normal (≤35 g/L)	38.5 (9.6–150.5)	*< 0.001*
Elevated (> 35 g/L)	61.4 (17.4–239.6)	
INR		
Normal (≤1.2)	54.5 (9.6–239.6)	*< 0.0001*
Elevated (> 1.2)	29.5 (10.3–138.8)	
Thrombocytes		
Normal (> 150/nL)	53 (9.6–239.6)	*< 0.001*
Reduced (≤150/nL)	33 (11.5–150.5)	
Treatment modality (first-line)		
TACE	40.9 (10.6–138.8))	*< 0.001*
Resection	61.7 (30.8–131.8)	
Liver transplantation	31.5 (23.2–125.6)	
Sorafenib	54.6 (13.8–239.6)	
Best supportive care	29.6 (9.6–150.5)	
Others (SIRT, RFA)	85.4 (29.4–104.6)	

Abbreviations: *IGF-1* insulin-like growth factor 1, *BCLC* Barcelona Clinic Liver Cancer, *α-FP* α-fetoprotein, *ALT* alanine transaminase, *AST* aspartate transaminase, *INR* international normalized ratio, *TACE* transarterial chemoembolization, *SIRT* selective internal radiation therapy, *RFA* radiofrequency ablation, *p*<0.05 was considered as significant

**Table 3:** Comparison of scoring systems and overall survival

Scoring system	Grade	n (%)	IGF-1 level (ng/mL)	Death events	Median OS	*p*
			median (range)		months (95% Cl)	
CTP score	A	108 (50.0)	60.5 (17.4–239.6)	79	12.6 (9.5–22.8)	*0.03*
	B	70 (32.4)	38.5 (10.6–138.8)	58	7.4 (5.3–11.1)	
	C	38 (17.6)	22.5 (9.6–58.5)	28	5.83 (3.8–11.6)	
IGF-CTP score	A	79 (36.6)	70.8 (29.4–239.6)	57	12.3 (9.5–28.6)	*0.04*
	B	67 (31.0)	41.7 (9.6–138.8)	57	7.7 (6.5–12.0)	
	C	70 (32.4)	24.2 (10.3–92.4)	51	6.8 (4.5–13.1)	

The log-rank test was used to compare overall survival

Abbreviations: *CTP* Child-Turcotte-Pugh, *IGF* insulin-like growth factor, *OS* overall survival, *CI* confidence interval, *p*<0.05 was considered as significant

### The amount of IGF-1 allows for only limited conclusions about the aggressiveness of HCC

Considering tumor characterization, there were no correlations between IGF-1 levels and tumor differentiation (grading) following the Edmondson–Steiner classification, multifocality, distant metastasis, or tumor size (Table 2). Serum alpha-fetoprotein level showed a negative correlation with IGF-1 levels without reaching statistical significance.

Next, we examined IGF-1 levels related to the BCLC scoring system. Most of our patients were categorized as BCLC stage C (*n* = 111, 51.4%) (Table 1). The highest IGF-1 levels were found in BCLC stage A and the lowest in BCLC stage D. IGF-1 measurements revealed a significant difference only between BCLC D and BCLC A, B, and C (*p* < 0.01) (Table 2)*.* Of interest, there was a significant correlation between vascular invasion by the tumor and amount of IGF-1. Patients with vascular invasion showed lower IGF-1 levels compared to patients with no vascular invasion (median (range) 35.8 ng/mL (9.6–138.8 ng/mL) vs. 45.8 ng/mL (11.5–239.6 ng/mL); *p* < 0.001) (Table 2).

### Differences in treatment and OS in dependence of IGF-1

The most common primary treatment for HCC in our cohort was TACE, performed in 40.3% of patients, followed by systemic therapy with sorafenib in 20.4%. Resection was performed in 12.5%, while 2.3% of patients underwent orthotropic liver transplantation as a first-line treatment. The remainder received best supportive care (BSC, 21.3%) or other therapies (3.2%) (Table 1). In patients with an IGF-1 level above the median of the cohort (> 43.4 ng/mL), significantly more resections were performed (21.4% vs. 2.9%; *p* < 0.001). In line with this, patients receiving BSC had IGF-1 levels below the median more often (32.7% vs. 10.7%; p < 0.01). Furthermore, patients undergoing liver resection had significantly higher IGF-1 values compared to those with BSC (p < 0.001) (Table 2). For all patients, the median OS was 11.4 months (range 0.5–118.2 months). Patients with IGF-1 levels above the cohort median had a significantly better prognosis than those with IGF-1 below the median (OS: 10.9 vs. 7.9 months; *p* < 0.05). Furthermore, comparison of IGF-1 between short-term survivors (1st quartile of time to death among deceased) and long-term (4th quartile of time to death among deceased) overall survival revealed a highly significant difference of IGF-1 levels (median IGF-1: 34.3 ng/mL vs. 54.8 ng/mL; *p* < 0.001).

### Reassignment of patients from traditional CTP score to IGF-CTP score

**Fig. 1:**
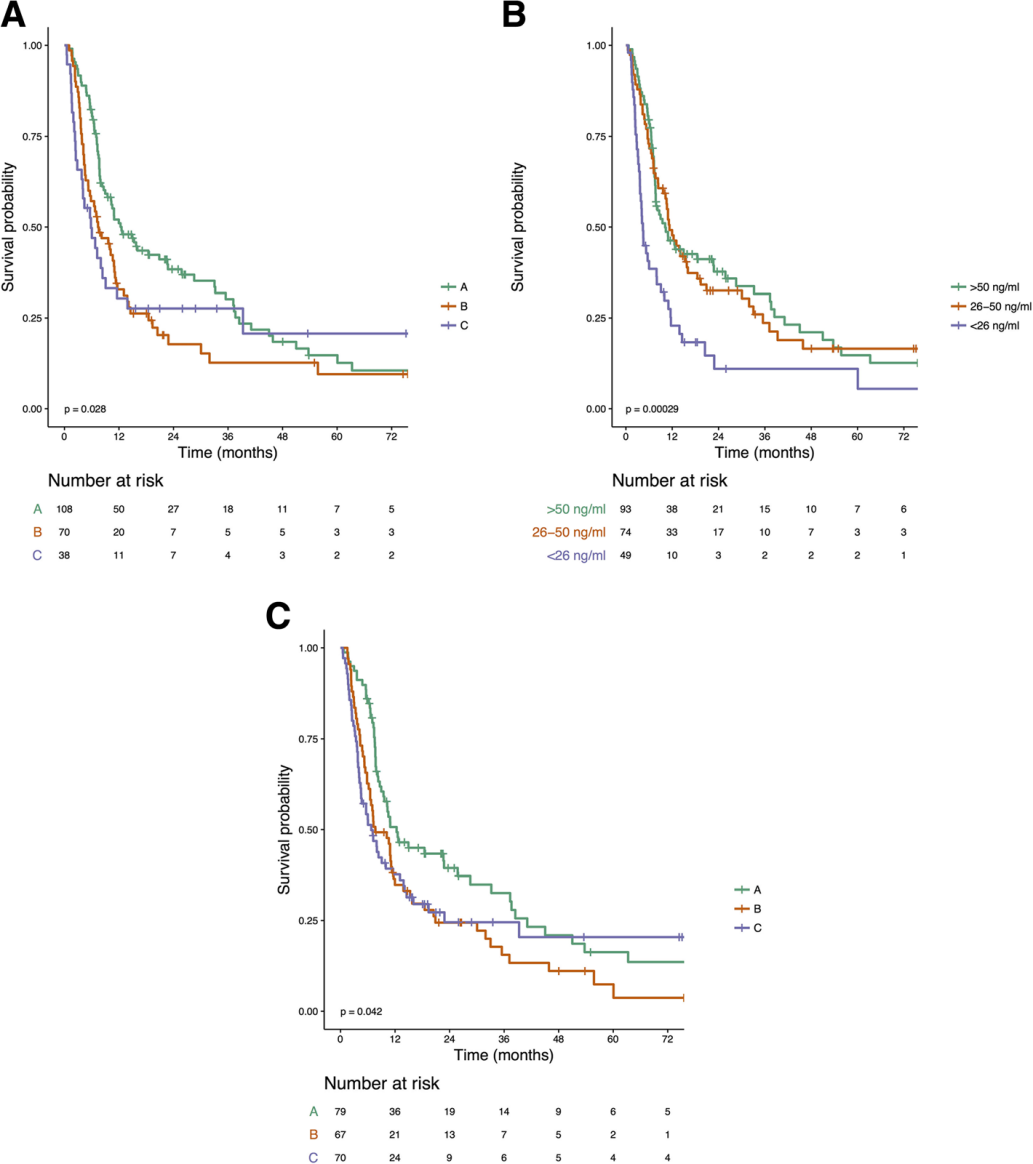
Kaplan–Meier survival curves of patients classified by CTP class (**a**), serum levels of IGF-1 (**b**), and IGF-CTP class (**c**). Tables below each graph show the numbers of patients at risk at various time points

CTP score as well as the IGF-CTP score stratified patients into low- (A), intermediate- (B), and high-risk (C) groups that differed in OS (p < 0.05). Most patients (*n* = 108, 50.0%) were classified as having a low-risk CTP score A and only 17.6% (*n* = 38) as having high-risk CTP score C. The IGF-CTP score stratified 79 patients (36.6%) into the low-risk group, followed by 32.4% (*n* = 70) in the high-risk group. Table 3 summarizes OS by IGF-1 levels and scoring system. In general, patients with high IGF-1 levels had a significantly better prognosis than those with low IGF-1 (*p* < 0.05). For 35.6% (*n* = 77) of patients, there was a difference between the original CTP class and IGF-CTP scoring system. With reassignment, however, stratification was no better regarding OS (Table 4). For example, 71 of 108 (65.7%) CTP-A patients were reclassified as IGF-CTP-A with a median OS of 12.7 months, while 29 (26.9%) were identified as IGF-CTP-B (AB group) with a median OS of 12.0 months, and 8 (7.4%) as IGF-CTP-C (AC group) with the worst prognosis (median OS 7.5 months). Patients in the original CTP A group who were reclassified as IGF-CTP-B (AB group) had the same prognosis as other CTP A patients classified as IGF-CTP-A (hazard ratio = 1.23; 95% confidence interval (Cl): 0.75 to 2.01; *p* = 0.42) (Table 4). Kaplan–Meier survival curves according to the IGF-CTP score showed no significant difference compared to the original CTP score (Fig. 1). A C-index analysis demonstrated no advantage in prognostic stratification by IGF-CTP scoring system. The C-index for the new IGF-CTP classification system was 0.622 (95% CI: 0.556–0.689), slightly lower than the C-index for the CTP classification, which was 0.646 (95% CI: 0.575–0.717); *p* = 0.8. However, CIs were fairly wide, so no clear distinction between classifications in terms of C-index was possible.

**Table 4:** Reclassification of scoring systems and overall survival

Scoring grades	Patients (n)	Death events (n)	Median OS months (95% Cl)	
Original A to new A (AA)	71	50	12.7 (10.3–33.2)	
Original A to new B (AB)	29	23	12.0 (7.1–37.2)	
Original A to new C (AC)	8	6	7.5 (6.0–NA)	
Original B to new A (BA)	8	7	7.9 (7.4–NA)	
Original B to new B (BB)	34	30	8.7 (5.8–11.7)	
Original B to new C (BC)	28	21	5.2 (3.9–NA)	
Original C to new A (CA)	0	0	NA	
Original C to new B (CB)	4	4	2.3 (1.5–NA)	
Original C to new C (CC)	34	24	6.8 (4.1–39.3)	
Scoring grades			HR (95%Cl)	*p*
AA			1.00 (referent)	
AB			1.23 (0.75–2.01)	0.42
AC			1.33 (0.57–3.10)	0.51
BA			1.19 (0.53–2.68)	0.67
BB			1.76 (1.12–2.78	*0.015*
BC			1.71 (1.02–2.85)	*0.04*
CB			9.58 (3.37–27.28)	*< 0.001*
CC			1.43 (0.88–2.33)	0.15
BB			1.00 (referent)	
AA			0.57 (0.36–0.90)	*0.015*
AB			0.70(0.40–1.20)	0.19
AC			0.75 (0.31–1.81)	0.53
BA			0.68 (0.29–1.58)	0.37
BC			0.97 (0.55–1.70)	0.91
CB			5.44 (1.88–15.78)	*0.002*
CC			0.81 (0.47–1.39)	0.45
CC			1.00 (referent)	
AA			0.70 (0.43–1.14)	0.15
AB			0.86 (0.48–1.52)	0.6
AC			0.93 (0.38–2.27)	0.87
BA			0.84 (0.35–1.98)	0.68
BB			1.23 (0.72–2.11)	0.45
BC			1.2 (0.66–2.15)	0.55
CB			6.7 (2.27–19.81)	*< 0.001*

The log-rank test was used to compare overall survival, *p*<0.05 was considered as significant

Abbreviations: *OS* overall survival, CI confidence interval, *NA* not applicable, i.e., upper bound cannot be computed

## Discussion

In this study, we validated serum IGF-1 as a marker for prediction in patient risk stratification and OS in HCC. However, replacing the two subjective variables encephalopathy and ascites with IGF-1 did not lead to more precise predictions compared to the original CTP classification in a cohort of European HCC patients.

Low serum levels of IGF-1 are common features in patients with diseased liver compared to healthy people [8] and in liver cirrhosis [8, 11, 21]. Here, advanced-stage liver cirrhosis (CHILD B/C) resulted in lower levels of IGF-1 compared to patients without liver cirrhosis or in CHILD A stage. Furthermore, serum IGF-1 levels were significantly lower in patients with HCC developing in cirrhosis compared with non-cirrhotic HCC. Recent studies recommended IGF-1 as a “surrogate marker for assessment of liver dysfunction” [8]. In the present study, patients with low serum levels of IGF-1 (< 43.4 ng/mL) had a significantly worse OS. Serum IGF-1 levels were associated with ALT and AST levels, bilirubin, albumin, INR, platelet count, and MELD score. Kaseb et al. found similar correlations with CTP score and bilirubin and the strongest correlation with AST level [14]. Tumor characteristics such as tumor differentiation, multinodularity, distant metastasis, and tumor size seemed not to influence the release of IGF-1 in our cohort. However, vascular invasion correlated with the amount of IGF-1. Kaseb et al., in their study with 288 HCC patients, reported results that are partly in contrast, with significant correlations between IGF-1 levels and number of tumor nodules and tumor size. Similar to our results, however, they found no association with tumor differentiation and distant metastasis [14]. Liu and colleagues also found significant associations with tumor size and number [22].

The median OS of the present analysis was 11.4 months. Similar survival data have been obtained by Di Costanzo et al. in an Italian cohort comprising 279 patients with sorafenib-treated advanced HCC with a median OS of 10.8 months [23] and in another study with 288 HCC patients with a median OS of 13.6 months [14]. In a recently published cohort from our department, including 1119 patients with HCC treated in an 11-year period, the median survival of all patients was 15.3 months [24].

In the present study, mean IGF-1 level was 52.4 ng/mL (standard deviation (SD) ± 35.33 ng/mL). Wang et al. reported in their meta-analysis of 20 studies published 2000–2016, including 432 patients with HCC, a mean serum IGF-1 value of 102.91 ng/mL (SD ± 85.89 ng/mL) [25]. In a subgroup analysis, an IGF-1 level above the median of the cohort (> 42.3 ng/mL) was tied to a better prognosis than IGF-1 values below the median. Therefore, serum IGF-1 level can be defined as a good parameter to evaluate patient risk. Further long-term research should address the predictive value of IGF-1 during chronic liver disease and different treatment strategies.

The proposed improvement of the modified CTP classification system (IGF-CTP) compared to traditional CTP is the replacement of subjective parameters (ascites, encephalopathy) with an objective parameter. This fact makes IGF-CTP a totally objective score, based solely on laboratory results, which excludes the variable of expertise in the evaluating physician or center during the assessment of patients with liver disease.

IGF-CTP was also used to predict survival in patients with HCC compared to the original CTP classification [17]. In 100 Egyptian patients, the IGF-CTP score was validated as a better survival predictor, with 32.5% of CTP A patients reclassified as IGF-CTP class B with significantly shorter OS than patients reclassified as IGF-CTP class A [26]. In a cohort with 393 Korean patients with HCC, mostly with underlying chronic viral hepatitis B, the IGF-CTP classification system showed no statistically significant improvement of stratification but demonstrated a trend towards better prediction of survival. In that analysis, only 14% of patients showed a difference between IGF-CTP class and CTP class [27]. In our cohort, 35.6% of patients were reclassified when using IGF-CTP. In both scoring systems, most patients (50.0 and 36.6%) were classified into low-risk group A. Although only 17.6% of patients were stratified into high-risk CTP score C, the IGF-CTP score allocated 36.6% of patients into high-risk group C. However, this reassignment did not improve prediction regarding OS. Consequently, the C-index analysis showed no relevant improvement in prediction. Reasons for the differing results regarding the prediction of the new IGF-CTP classification system might be the characteristics of the underlying cohorts (Table 5).

**Table 5:** Literature overview of studies analyzing CTP-IGF scoring system

Cohort	Mean age ± SD (years)	Male sex (%)	Viral hepatitis (%)	Liver cirrhosis (%)	CTP class (%)			BCLC stage (%)					OS (months)
					A	B	C	0	A	B	C	D	
US training [17] *n* = 310	62.6 ± 11.8	70.3	44.8	62.6	71.2	25.5	2.6	6.5	8.7	9.7	63.2	7.4	13.2
US validation [17] *n* = 155	63.2 ± 10.8	72.9	50.3	63.6	81.3	16.1	2.6	1.3	8.4	11.0	76.8	2.5	15.7
Egyptian [26] *n* = 100	56.7 ± 8.7	83.0	100.0	87.0	40.0	32.0	28.0	0.0	1.0	8.0	60.0	31.0	8.6
Korean [27] *n* = 393	56.8 ± 9.5	77.9	91.1	48.9	85.0	14.5	0.5	20.9	40.2	9.4	29.0	0.5	Missing
German n = 216	67.6 ± 9.5	86.1	14.6	81.3	50.0	32.4	17.6	0.0	16.7	11.6	51.4	20.4	11.4

In all following studies, the same assay for quantification of IGF-1 was used. The IGF-CTP classification was developed and validated in two US cohorts where most patients had viral hepatitis as the underlying liver disease for HCC [14, 17]. In the Egyptian validation study, all patients had viral-induced HCC [26], as was also the case in the Korean cohort, especially HBV (78.9%) [27] (Table 5). In contrast, our cohort had alcohol-induced liver disease as the most frequent underlying cause of HCC in 44.4% and viral hepatitis in only 24.6% of cases.

Another difference between the present cohort and recent studies is the proportion of cirrhosis in the study population. In the US cohorts, liver cirrhosis was present in 62.6 and 63.6% of patients [14, 17], whereas 48.9% of patients showed cirrhosis in the Korean study [27]. Only the Egyptian cohort had a similar proportion of cirrhosis as in our study, with 87% of patients in comparison to our study’s rate of 80.1% [26]. Furthermore, the classification of patients into CTP risk stratification varied between the different cohorts. In previous validation studies, most patients were in low-risk groups, while only 0.5–2.6% of patients were stratified into the high-risk group CTP C. The Egyptian cohort was an exception, with 28.0% of patients in CTP C. In our cohort, 17.6% of patients were allocated to the high-risk group CTP C.

The mean age of the present study population was 67.6 years, which represents the oldest cohort compared to others studies analyzing IGF-1 levels (Table 5). However, since the incidence peak of HCC is supposed to be at 70 years in Europe according to the latest EASL guidelines [28], our cohort can generally be considered representative. Nonetheless the age difference limits comparability of the current results with previous IGF-1 analyses.

In conclusion, our European cohort differs from other published validation studies in terms of presenting different underlying liver diseases, a higher proportion of patients with cirrhosis, and consequently less hepatic reserve. Although viral hepatitis is the leading cause for HCC globally, in the western world, alcohol abuse is one of the leading etiologies [29]. It is estimated that 18 to 33% of the total number of HCCs is caused by past and present alcohol intake in several European countries [29]. Recently, Karageorgos and colleagues showed a change in incidence and risk factors for cirrhosis and HCC in Crete, with significantly decreased HCV association and alcohol as the top-ranked risk factor [30]. In a German cohort of 458 HCC patients, chronic alcohol abuse was identified as the leading risk factor in 57.2% [31]. Especially in Europe, alcohol abuse is becoming an important risk factor in HCC, and it thus is essential to validate the new IGF-CTP classification system in a non-viral hepatitis cohort. Because the majority of HCC (80 to 90%) develops in the setting of cirrhosis, it also seems to be important to validate the new prognostic score in a cohort with a high rate of liver cirrhosis.

## Conclusion

This study is the first to investigate IGF-1 and the IGF-CTP classification in a European cohort of HCC patients. The IGF-CTP classification was not superior to the original CTP classification for predicting patient survival and liver function. Serum IGF-1 level correlated with several clinical factors and is a prognostic marker for risk stratification and OS. In summary, IGF-1 might serve as a useful additional parameter for patient risk stratification in the future. However, inclusion of the IGF-CTP score currently offers no advantage in comparison to CTP in a European cohort.

## Abbreviations

AFP: α-fetoprotein
BCLC: Barcelona clinic liver cancer staging system
BSC: best supportive care
CTP: Child-Turcotte-Pugh score
HCC: hepatocellular carcinoma
MELD: model of end-stage liver disease
NASH: non-alcoholic steatohepatitis
OLT: orthotopic liver transplantation
OS: overall survival
PBC: primary biliary cholangitis
PSC: primary sclerosing cholangitis
RFA: radiofrequency ablation
SIRT: selective internal radiation therapy
TACE: transarterial chemoembolization
UICC: Union Internationale Contre le Cancer
